# Supplementary material for: Polygenic risk score for obesity and the quality, quantity, and timing of workplace food purchases: A secondary analysis from the ChooseWell 365 randomized trial
Source: PLoS Med. 2020 Jul 21;17(7):e1003219. doi: 10.1371/journal.pmed.1003219 (PMC7373257; doi:10.1371/journal.pmed.1003219)
Supplement: S1 Text — (DOCX) [file pmed.1003219.s002.docx]

**S1 Text: Prospective Analysis Plan**

**for “ChooseWell 365” baseline genetic association testing with workplace food purchases**

**Pre-imputation genotype QC criteria:**

Align genotype data to hg19 forward strand genome build

Sample QC

- Sample call rate (cut off >95% threshold recommended)
- Exclude samples with heterozygosity > median + 3*IQR
- Remove gender mismatches
- Remove duplicates
- Remove PCA outliers using a PCA projection of the study samples onto 1KG reference samples (exclude detected relatives).

SNP QC

- Hardy-Weinberg p>10^-6^, SNP call rate ≥98%
- Remove monomorphic markers

**Imputation and post-imputation QC criteria:**

- Impute using Michigan imputation server (HRC panel)
- Imputation quality
- Minor allele freq cutoff (>0.01)

## **Genetic Risk Score for body mass index**

- Score is weighted by effect estimate from discovery GWAS^1^
- Global GRS (genome-wide polygenic score)^1^ – higher score = higher BMI
- BMI GRS (97 known loci)^1^ – higher score = higher BMI
- CNS BMI GRS (54/97 known loci)^1^ – higher score = higher BMI
- Non-CNS BMI GRS (43/97 known loci)^1^ – higher score = higher BMI
- Use PRSice/PLINK to create genetic risk score for each participant
- Missing SNPs from an individual; allow for missing SNPs and weight it;
  if >10% missingness of SNPs

**Baseline phenotypes and covariates**

- Measures:
  - BMI: measured at baseline
  - Purchases: healthy purchasing score, total/food/beverage purchases, timing of breakfast purchase and lunch purchase
  - Self-report: breakfast/lunch/dinner skipping; breakfast/lunch/dinner at home
- Covariates: Age, gender, genotypic array, PCs
- Inverse normalize skewed variables

**Primary Baseline Association Analyses:**

1. Association of genetic risk scores with baseline BMI
2. Association of BMI GRS with cafeteria workplace purchases
3. Association of BMI GRS with self-report dietary outcomes
   1. **Covariates:** all genetic analyses adjusted for age, sex, seasonality, genotyping array, and PCs

**References**

1. Locke AE, Kahali B, Berndt SI, et al. Genetic studies of body mass index yield new insights for obesity biology. *Nature*. 2015;518(7538):197-206. doi:10.1038/nature14177
