# Supplementary material for: Polygenic risk score for obesity and the quality, quantity, and timing of workplace food purchases: A secondary analysis from the ChooseWell 365 randomized trial
Source: PLoS Med. 2020 Jul 21;17(7):e1003219. doi: 10.1371/journal.pmed.1003219 (PMC7373257; doi:10.1371/journal.pmed.1003219)
Supplement: S1 Table — BMI, body mass index. (DOCX) [file pmed.1003219.s003.docx]

**S1 Table.** Individual 97 BMI single nucleotide polymorphism associations with BMI (in kg/m^2^) in the “ChooseWell 365” study (*n* =397).

| **SNP** | **Chr:position** | **Nearest Gene(s)** | **Alleles**  **(E/A)** | **EAF** | **Beta (SE)** | ***P*** |
| --- | --- | --- | --- | --- | --- | --- |
|  | **Variants highly expressed in the CNS^1^** | | | | | |
| rs12286929 | 11:115022404 | *CADM1* | G/A | 0.53 | 1.16 (0.44) | 0.02 |
| rs7164727 | 15:73093991 | *LOC100287559; BBS4* | T/C | 0.69 | 1.13 (0.45) | 0.03 |
| rs10182181 | 2:25150296 | *NCOA1, SH2B1, APOBR* | G/A | 0.45 | 1.03 (0.42) | 0.01 |
| rs10968576 | 9:28414339 | *LINGO2* | G/A | 0.29 | 0.97 (0.47) | 0.03 |
| rs9914578 | 17:2005136 | *SMG6; N29617* | G/C | 0.19 | 0.94 (0.55) | 0.14 |
| rs2287019 | 19:46202172 | *QPCTL, GIPR* | C/T | 0.81 | 0.86 (0.54) | 0.09 |
| rs11030104 | 11:27684517 | *BDNF* | A/G | 0.81 | 0.76 (0.56) | 0.20 |
| rs11057405 | 12:122781897 | *CLIP1* | G/A | 0.92 | 0.75 (0.80) | 0.62 |
| rs7138803 | 12:50247468 | *BCDIN3D, FAIM2, ADCY3, POMC* | A/G | 0.37 | 0.71 (0.46) | 0.13 |
| rs3849570 | 3:81792112 | *GBE1* | A/C | 0.35 | 0.62 (0.46) | 0.19 |
| rs7141420 | 14:79899454 | *NRXN3* | T/C | 0.53 | 0.58 (0.46) | 0.41 |
| rs7599312 | 2:213413231 | *ERBB4* | G/A | 0.74 | 0.53 (0.47) | 0.26 |
| rs1558902 | 16:53803574 | *FTO* | A/T | 0.41 | 0.52 (0.45) | 0.47 |
| rs7239883 | 18:40147671 | *LOC284260; RIT2* | G/A | 0.39 | 0.48 (0.42) | 0.22 |
| rs6567160 | 18:57829135 | *MC4R* | C/T | 0.25 | 0.45 (0.52) | 0.41 |
| rs3810291 | 19:47569003 | *ZC3H4* | A/G | 0.68 | 0.41 (0.47) | 0.30 |
| rs10733682 | 9:129460914 | *LMX1B* | A/G | 0.45 | 0.33 (0.45) | 0.77 |
| rs9400239 | 6:108977663 | *FOXO3; HSS00296402* | C/T | 0.69 | 0.30 (0.48) | 0.63 |
| rs2836754 | 21:40291740 | *ETS2* | C/T | 0.62 | 0.28 (0.44) | 0.67 |
| rs4740619 | 9:15634326 | *C9orf93* | T/C | 0.51 | 0.27 (0.42) | 0.64 |
| rs12401738 | 1:78446761 | *FUBP1, USP33* | A/G | 0.35 | 0.23 (0.44) | 0.64 |
| rs9925964 | 16:31129942 | *KAT8;ZNF646; VKORC1;ZNF668;* | A/G | 0.62 | 0.21 (0.43) | 0.50 |
| rs13078960 | 3:85807590 | *CADM2* | G/T | 0.21 | 0.21 (0.55) | 0.62 |
| rs4256980 | 11:8673939 | *TRIM66, TUB* | G/C | 0.62 | 0.18 (0.45) | 0.59 |
| rs3888190 | 16:28889486 | *ATXN2L, SBK1, SULT1A2, TUFM* | A/C | 0.37 | 0.18 (0.45) | 0.61 |
| rs3101336 | 1:72751185 | *NEGR1* | C/T | 0.63 | 0.18 (0.45) | 0.83 |
| rs13201877 | 6:137675541 | *IFNGR1; OLIG3* | G/A | 0.14 | 0.17 (0.63) | 0.97 |
| rs16951275 | 15:68077168 | *MAP2K5, LBXCOR1, MTCH2, C1QTNF4,* | T/C | 0.74 | 0.14 (0.51) | 0.72 |
| rs4787491 | 16:30015337 | *MAPK3; KCTD13; INO80E;TAOK2;* | G/A | 0.54 | 0.12 (0.44) | 0.62 |
| rs12885454 | 14:29736838 | *PRKD1* | C/A | 0.66 | 0.09 (0.45) | 0.75 |
| rs11583200 | 1:50559820 | *ELAVL4* | C/T | 0.39 | 0.07 (0.43) | 0.85 |
| rs17094222 | 10:102395440 | *HIF1AN* | C/T | 0.24 | 0.03 (0.52) | 0.94 |
| rs2820292 | 1:201784287 | *NAV1* | C/A | 0.54 | 0.01 (0.45) | 0.78 |
| rs13021737 | 2:632550 | *TMEM18* | G/A | 0.79 | -0.02 (0.53) | 0.96 |
| rs7243357 | 18:56883319 | *GRP* | T/G | 0.8 | -0.02 (0.53) | 0.43 |
| rs2080454 | 16:49062590 | *CBLN1* | C/A | 0.34 | -0.06 (0.45) | 0.71 |
| rs17405819 | 8:76806584 | *HNF4G* | T/C | 0.7 | -0.07 (0.47) | 0.56 |
| rs11165643 | 1:96924097 | *PTBP2* | T/C | 0.58 | -0.08 (0.44) | 0.39 |
| rs1808579 | 18:21104888 | *NPC1, C18orf8* | C/T | 0.53 | -0.13 (0.43) | 0.91 |
| rs1167827 | 7:75163169 | *HIP1; PMS2L3; PMS2P5;WBSCR16* | G/A | 0.58 | -0.13 (0.44) | 0.60 |
| rs10132280 | 14:25928179 | *STXBP6* | C/A | 0.71 | -0.15 (0.48) | 0.49 |
| rs6804842 | 3:25106437 | *RARB* | G/A | 0.55 | -0.16 (0.42) | 0.76 |
| rs29941 | 19:34309532 | *KCTD15* | G/A | 0.67 | -0.19 (0.46) | 0.75 |
| rs11126666 | 2:26928811 | *KCNK3* | A/G | 0.26 | -0.21 (0.48) | 1.00 |
| rs9540493 | 13:66205704 | *MIR548X2; PCDH9* | A/G | 0.45 | -0.32 (0.43) | 0.51 |
| rs1516725 | 3:185824004 | *ETV5* | C/T | 0.86 | -0.37 (0.62) | 0.56 |
| rs492400 | 2:219349752 | *PLCD4; CYP27A1; USP37; TTLL4;* | C/T | 0.41 | -0.39 (0.45) | 0.16 |
| rs7899106 | 10:87410904 | *GRID1* | G/A | 0.05 | -0.43 (1.01) | 0.67 |
| rs12446632 | 16:19935389 | *GPRC5B, IQCK* | G/A | 0.87 | -0.45 (0.62) | 0.47 |
| rs13191362 | 6:163033350 | *PARK2* | A/G | 0.9 | -0.45 (0.71) | 0.58 |
| rs2033529 | 6:40348653 | *TDRG1; LRFN2* | G/A | 0.26 | -0.54 (0.50) | 0.23 |
| rs2075650 | 19:45395619 | *TOMM40, APOE, APOC1* | A/G | 0.87 | -0.61 (0.66) | 0.40 |
| rs10938397 | 4:45182527 | *GNPDA2, GABRG1* | G/A | 0.44 | -0.74 (0.45) | 0.18 |
| rs3736485 | 15:51748610 | *SCG3; DMXL2* | A/G | 0.47 | -0.93 (0.45) | 0.03 |
|  | **Variants highly expressed in other tissues^2^** | | | | | |
| rs17024393 | 1:110154688 | *GNAT2, AMPD2* | C/T | 0.03 | 1.59 (1.33) | 0.26 |
| rs13107325 | 4:103188709 | *SLC39A8* | T/C | 0.07 | 1.52 (0.88) | 0.11 |
| rs2112347 | 5:75015242 | *POC5, HMGCR, COL4A3BP* | T/G | 0.64 | 1.34 (0.43) | 0.003 |
| rs1000940 | 17:5283252 | *RABEP1* | G/A | 0.32 | 1.13 (0.46) | 0.04 |
| rs12940622 | 17:78615571 | *RPTOR* | G/A | 0.57 | 0.84 (0.45) | 0.08 |
| rs11847697 | 14:30515112 | *PRKD1* | T/C | 0.05 | 0.75 (0.99) | 0.45 |
| rs758747 | 16:3627358 | *NLRC3* | T/C | 0.27 | 0.56 (0.49) | 0.12 |
| rs2033732 | 8:85079709 | *RALYL* | C/T | 0.76 | 0.55 (0.52) | 0.47 |
| rs17203016 | 2:208255518 | *CREB1; KLF7* | G/A | 0.19 | 0.52 (0.55) | 0.18 |
| rs543874 | 1:177889480 | *SEC16B* | G/A | 0.19 | 0.46 (0.59) | 0.65 |
| rs2245368 | 7:76608143 | *PMS2L11* | C/T | 0.17 | 0.40 (0.56) | 0.18 |
| rs9641123 | 7:93197732 | *CALCR; hsa-miR-653* | C/G | 0.42 | 0.37 (0.43) | 0.31 |
| rs17724992 | 19:18449238 | *GDF15; PGPEP1* | A/G | 0.73 | 0.37 (0.51) | 0.69 |
| rs7903146 | 10:114758349 | *TCF7L2* | C/T | 0.68 | 0.36 (0.46) | 0.48 |
| rs1016287 | 2:59305625 | *LINC01122* | T/C | 0.30 | 0.35 (0.48) | 0.36 |
| rs1928295 | 9:120378483 | *TLR4* | T/C | 0.56 | 0.34 (0.46) | 0.72 |
| rs12016871 | 13:28017782 | *MTIF-GTF3A* | T/C | 0.19 | 0.29 (0.56) | 0.46 |
| rs11191560 | 10:104869038 | *NT5C2; CYP17A1; SFXN2* | C/T | 0.09 | 0.29 (0.77) | 0.80 |
| rs2176040 | 2:227092802 | *LOC646736; IRS1* | A/G | 0.36 | 0.25 (0.45) | 0.42 |
| rs1460676 | 2:164567689 | *FIGN* | C/T | 0.17 | 0.24 (0.58) | 0.46 |
| rs12566985 | 1:75002193 | *FPGT-TNNI3K* | G/A | 0.42 | 0.11 (0.44) | 0.84 |
| rs2121279 | 2:143043285 | *LRP1B* | T/C | 0.14 | 0.07 (0.62) | 0.94 |
| rs3817334 | 11:47650993 | *CELF1* | T/C | 0.41 | 0.04 (0.43) | 0.84 |
| rs2176598 | 11:43864278 | *HSD17B12* | T/C | 0.25 | -0.02 (0.50) | 0.55 |
| rs2207139 | 6:50845490 | *TFAP2B* | G/A | 0.20 | -0.04 (0.56) | 0.89 |
| rs657452 | 1:49589847 | *AGBL4* | A/G | 0.39 | -0.07 (0.45) | 0.56 |
| rs12429545 | 13:54102206 | *OLFM4* | A/G | 0.14 | -0.11 (0.61) | 0.84 |
| rs16851483 | 3:141275436 | *RASA2* | T/G | 0.06 | -0.11 (0.89) | 0.87 |
| rs11688816 | 2:63053048 | *EHBP1* | G/A | 0.52 | -0.12 (0.45) | 0.68 |
| rs1528435 | 2:181550962 | *UBE2E3* | T/C | 0.60 | -0.13 (0.42) | 0.79 |
| rs2650492 | 16:28333411 | *SBK1; APOBR* | A/G | 0.28 | -0.14 (0.47) | 0.55 |
| rs977747 | 1:47684677 | *TAL1* | T/G | 0.42 | -0.16 (0.45) | 0.66 |
| rs2365389 | 3:61213993 | *FHIT* | C/T | 0.64 | -0.17 (0.46) | 0.62 |
| rs17001654 | 4:77129568 | *NUP54; SCARB2* | G/C | 0.18 | -0.17 (0.57) | 0.68 |
| rs205262 | 6:34563164 | *C6orf106, SNRPC* | G/A | 0.27 | -0.22 (0.52) | 0.64 |
| rs6465468 | 7:95169514 | *ASB4* | T/G | 0.26 | -0.23 (0.5) | 0.95 |
| rs16907751 | 8:81375457 | *ZBTB10* | C/T | 0.89 | -0.27 (0.73) | 0.62 |
| rs9374842 | 6:120185665 | *LOC285762;* | T/C | 0.74 | -0.31 (0.50) | 0.45 |
| rs6091540 | 20:51087862 | *ZFP64* | C/T | 0.68 | -0.36 (0.48) | 0.73 |
| rs7715256 | 5:153537893 | *GALNT10* | G/T | 0.43 | -0.37 (0.44) | 0.26 |
| rs11727676 | 4:145659064 | *HHIP* | T/C | 0.90 | -0.38 (0.77) | 0.63 |
| rs6477694 | 9:111932342 | *EPB41L4B; C9orf4* | C/T | 0.34 | -0.46 (0.46) | 0.49 |
| rs1441264 | 13:79580919 | *MIR548A2* | A/G | 0.59 | -1.18 (0.44) | 0.01 |

^1^54 loci near genes enriched for expression in regions of the CNS, including hypothalamus circuits that regulate appetite. ^2^43 loci near genes enriched for expression in other tissues with unlikely CNS functions. Genetic association analyses were performed using PLINK and an additive genetic model adjusted for age, sex, and 5 principal components of ancestry. Nearest Gene(s) column indicates all genes within the locus of interest. Beta (SE) estimates are per each additional effect (i.e., BMI-increasing) allele in kg/m^2^.

**Abbreviations:** Chr, chromosome; CNS, central nervous system; E/A, effect/alternative alleles; position, base pair coordinate hg19; SNP, single nucleotide polymorphism.
