# Supplementary material for: Polygenic risk score for obesity and the quality, quantity, and timing of workplace food purchases: A secondary analysis from the ChooseWell 365 randomized trial
Source: PLoS Med. 2020 Jul 21;17(7):e1003219. doi: 10.1371/journal.pmed.1003219 (PMC7373257; doi:10.1371/journal.pmed.1003219)
Supplement: S2 Table — BMI, body mass index. (DOCX) [file pmed.1003219.s004.docx]

**S2 Table.** Unadjusted (*P_unadj_*) and false discovery rate corrected (*P*_adj_) *P* values for BMI genetic scores associations with workplace purchases and self-reported meal skipping and meals prepared at home.

|  |  | **BMI_GPS_** | |  | **BMI_97_** | |  | **BMI_CNS_** | |  | **BMI_non-CNS_** | |
| --- | --- | --- | --- | --- | --- | --- | --- | --- | --- | --- | --- | --- |
|  |  | *P_unadj_* | *P_adj_* |  | *P_unadj_* | *P_adj_* |  | *P_unadj_* | *P_adj_* |  | *P_unadj_* | *P_adj_* |
| **Workplace purchases** |  |  |  |  |  |  |  |  |  |  |  |  |
| Healthy purchasing score, % |  | 0.015 | 0.059 |  | 0.953 | 0.953 |  | 0.901 | 0.901 |  | 0.954 | 0.954 |
| Total purchases, units |  | 0.161 | 0.194 |  | 0.561 | 0.841 |  | 0.044 | 0.133 |  | 0.223 | 0.445 |
| Food purchases, units |  | 0.029 | 0.059 |  | 0.926 | 0.953 |  | 0.044 | 0.133 |  | 0.082 | 0.245 |
| Beverage purchases, units |  | 0.965 | 0.965 |  | 0.291 | 0.841 |  | 0.293 | 0.586 |  | 0.629 | 0.755 |
| Breakfast timing, minutes |  | 0.030 | 0.059 |  | 0.101 | 0.608 |  | 0.789 | 0.901 |  | 0.009 | 0.057 |
| Lunch timing, minutes |  | 0.120 | 0.180 |  | 0.430 | 0.841 |  | 0.819 | 0.901 |  | 0.450 | 0.675 |
| **Self-reported** |  |  |  |  |  |  |  |  |  |  |  |  |
| Skip breakfast |  | 0.111 | 0.271 |  | 0.219 | 0.438 |  | 0.970 | 0.970 |  | 0.029 | 0.086 |
| Skip lunch |  | 0.740 | 0.740 |  | 0.491 | 0.644 |  | 0.507 | 0.970 |  | 0.424 | 0.630 |
| Skip dinner |  | 0.226 | 0.271 |  | 0.597 | 0.644 |  | 0.548 | 0.970 |  | 0.630 | 0.630 |
| Breakfast prepared at home |  | 0.180 | 0.271 |  | 0.099 | 0.438 |  | 0.924 | 0.970 |  | 0.043 | 0.086 |
| Lunch prepared at home |  | 0.215 | 0.271 |  | 0.644 | 0.644 |  | 0.122 | 0.730 |  | 0.010 | 0.062 |
| Dinner prepared at home |  | 0.032 | 0.190 |  | 0.204 | 0.438 |  | 0.866 | 0.970 |  | 0.545 | 0.630 |

Unadjusted (*P_unadj_*) *P* values are values reported in the main text and presented Figures 2—5. Based on the biological functions of genes in or near the 97 previously identified BMI loci, the BMI_CNS_ PRS and BMInon-CNS PRS are comprised of 54 variants previously classified as CNS-related and 43 variants previously classified as non-CNS-related, respectively.

**Abbreviations:** BMI, body mass index; CNS, central nervous system; GPS, genome-wide polygenic score.
