# Supplementary material for: Polygenic risk score for obesity and the quality, quantity, and timing of workplace food purchases: A secondary analysis from the ChooseWell 365 randomized trial
Source: PLoS Med. 2020 Jul 21;17(7):e1003219. doi: 10.1371/journal.pmed.1003219 (PMC7373257; doi:10.1371/journal.pmed.1003219)
Supplement: S3 Table — BMI, body mass index. (DOCX) [file pmed.1003219.s005.docx]

**S3 Table.** BMI genetic scores as continuous measures associations with workplace purchases and self-reported meal skipping and meals prepared at home.

|  |  | **BMI_GPS_** | |  | **BMI_97_** | |  | **BMI_CNS_** | |  | **BMI_non-CNS_** | |
| --- | --- | --- | --- | --- | --- | --- | --- | --- | --- | --- | --- | --- |
|  |  | Beta or OR  [95% CI] per  one-SD | *P* value |  | Beta or OR  [95% CI] per  risk allele | *P* value |  | Beta or OR  [95% CI] per  risk allele | *P* value |  | Beta or OR  [95% CI] per  risk allele | *P* value |
| **Workplace purchases** |  |  |  |  |  |  |  |  |  |  |  |  |
| Healthy purchasing score, % |  | -1.6 [-2.9, -0.3] | 0.02 |  | -0.1 [-0.3, 0.2] | 0.63 |  | -0.01 [-0.3, 0.3] | 0.90 |  | -0.16 [-0.6, 0.2] | 0.51 |
| Total purchases, units |  | 5.8 [-1.2, 12.8] | 0.11 |  | -0.3 [-1.4, 0.9] | 0.43 |  | -0.9 [-2.3, 0.5] | 0.12 |  | 1.1 [-0.95, 3.1] | 0.37 |
| Food purchases, units |  | 5.2 [0.1, 10.4] | 0.02 |  | -0.1 [-1, 0.7] | 0.72 |  | -0.7 [-1.7, 0.4] | 0.11 |  | 1.1 [-0.4, 2.6] | 0.09 |
| Beverage purchases, units |  | 0.6 [-2.6, 3.9] | 0.81 |  | -0.2 [-0.7, 0.4] | 0.50 |  | -0.2 [-0.9, 0.4] | 0.59 |  | -0.01 [-0.96, 0.9] | 0.71 |
| Breakfast timing, minutes |  | 3.2 [-1.6, 7.9] | 0.19 |  | 0.6 [-0.2, 1.4] | 0.13 |  | -0.05 [-0.99, 0.9] | 0.92 |  | 2.02 [0.6, 3.4] | 0.004 |
| Lunch timing, minutes |  | 1.5 [-1.5, 4.6] | 0.32 |  | 0.3 [-0.2, 0.8] | 0.23 |  | 0.2 [-0.4, 0.8] | 0.47 |  | 0.5 [-0.4, 1.4] | 0.31 |
| **Self-reported** |  |  |  |  |  |  |  |  |  |  |  |  |
| Skip breakfast |  | 1.19 [0.96, 1.5] | 0.12 |  | 1.03 [0.99, 1.06] | 0.16 |  | 0.995 [0.95, 1.04] | 0.82 |  | 1.09 [1.03, 1.17] | 0.01 |
| Skip lunch |  | 0.98 [0.79, 1.2] | 0.89 |  | 1.02 [0.98, 1.05] | 0.36 |  | 1.003 [0.96, 1.05] | 0.87 |  | 1.05 [0.98, 1.11] | 0.17 |
| Skip dinner |  | 1.16 [0.9, 1.5] | 0.26 |  | 1.02 [0.98, 1.06] | 0.37 |  | 0.996 [0.95, 1.05] | 0.87 |  | 1.07 [0.99, 1.15] | 0.08 |
| Breakfast prepared at home |  | 0.93 [0.76, 1.1] | 0.48 |  | 0.98 [0.95, 1.01] | 0.25 |  | 0.998 [0.96, 1.04] | 0.90 |  | 0.94 [0.89, 1.00] | 0.07 |
| Lunch prepared at home |  | 0.87 [0.7, 1.1] | 0.25 |  | 1.00 [0.96, 1.03] | 0.84 |  | 1.04 [0.99, 1.09] | 0.12 |  | 0.91 [0.85, 0.98] | 0.01 |
| Dinner prepared at home |  | 0.78 [0.59, 1.0] | 0.08 |  | 1.01 [0.97, 1.06] | 0.66 |  | 1.01 [0.96, 1.06] | 0.76 |  | 1.01 [0.93, 1.1] | 0.75 |

Associations results are adjusted betas or odds ratios and 95% confidence interval from multivariable linear or logistic regression models for PRS or GPS as continuous measures associations with workplace purchases and survey-derived meal habits adjusted for age, sex, seasonality, and the 5 principal components of ancestry. Higher purchasing score=healthier purchases (0-100%). Odds ratio >1 indicates more meal skipping or more meal prepared at home. Based on the biological functions of genes in or near the 97 previously identified BMI loci, the BMI_CNS_ PRS and BMInon-CNS PRS are comprised of 54 variants previously classified as CNS-related and 43 variants previously classified as non-CNS-related, respectively. *P* values are unadjusted for multiple testing.

**Abbreviations:** BMI, body mass index; CNS, central nervous system; GPS, genome-wide polygenic score; OR, odds ratio; SD, standard deviation.
