# Supplementary material for: Polygenic risk score for obesity and the quality, quantity, and timing of workplace food purchases: A secondary analysis from the ChooseWell 365 randomized trial
Source: PLoS Med. 2020 Jul 21;17(7):e1003219. doi: 10.1371/journal.pmed.1003219 (PMC7373257; doi:10.1371/journal.pmed.1003219)
Supplement: S4 Table — (DOCX) [file pmed.1003219.s006.docx]

**S4 Table.** Sensitivity analyses for BMI genetic scores associations with workplace purchases and self-reported meal skipping and meals prepared at home further adjusted for job type, education level, smoking status, and physical activity level.

|  |  | **BMI_GPS_** | |  | **BMI_97_** | |  | **BMI_CNS_** | |  | **BMI_non-CNS_** | |
| --- | --- | --- | --- | --- | --- | --- | --- | --- | --- | --- | --- | --- |
|  |  | Beta or OR  [95% CI] | *P* value |  | Beta or OR  [95% CI] | *P* value |  | Beta or OR  [95% CI] | *P* value |  | Beta or OR  [95% CI] | *P* value |
| **Workplace purchases** |  |  |  |  |  |  |  |  |  |  |  |  |
| Healthy purchasing score, % |  | -4.1 [-7.9, -0.3] | 0.04 |  | 0.5 [-3.3, 4.2] | 0.04 |  | -0.2 [-4.0, 3.5] | 0.04 |  | 0.7 [-3.0, 4.5] | 0.04 |
| Total purchases, units |  | 13.9 [-6.0, 33.8] | 0.18 |  | -4.1 [-23.8, 15.6] | 0.52 |  | -15.5 [-35.2, 4.1] | 0.07 |  | 12.1 [-7.6, 31.8] | 0.31 |
| Food purchases, units |  | 13.9 [-0.6, 28.3] | 0.03 |  | 1.2 [-13.1, 15.6] | 0.89 |  | -10 [-24.2, 4.3] | 0.08 |  | 8.9 [-5.4, 23.2] | 0.12 |
| Beverage purchases, units |  | 0.1 [-9.3, 9.5] | 0.92 |  | -5.3 [-14.6, 4.0] | 0.25 |  | -5.6 [-14.9, 3.7] | 0.33 |  | 3.2 [-6.1, 12.5] | 0.66 |
| Breakfast timing, minutes |  | 15.7 [1.9, 29.6] | 0.03 |  | 11 [-2.7, 24.8] | 0.03 |  | -1.9 [-15.7, 11.8] | 0.03 |  | 16.5 [2.8, 30.2] | 0.03 |
| Lunch timing, minutes |  | 4.4 [-4.3, 13.1] | 0.32 |  | 3.6 [-4.9, 12.2] | 0.32 |  | -0.6 [-9.2, 7.9] | 0.32 |  | 2.1 [-6.4, 10.7] | 0.32 |
| **Self-reported** |  |  |  |  |  |  |  |  |  |  |  |  |
| Skip breakfast |  | 1.7 [0.9, 3.2] | 0.13 |  | 1.4 [0.7, 2.7] | 0.35 |  | 1.0 [0.5, 1.9] | 0.99 |  | 1.9 [1.0, 3.7] | 0.05 |
| Skip lunch |  | 1.1 [0.6, 2.1] | 0.76 |  | 1.2 [0.7, 2.4] | 0.51 |  | 1.2 [0.7, 2.4] | 0.50 |  | 1.2 [0.6, 2.3] | 0.57 |
| Skip dinner |  | 1.5 [0.7, 3.4] | 0.31 |  | 1.2 [0.6, 2.5] | 0.69 |  | 1.1 [0.5, 2.5] | 0.79 |  | 1.2 [0.6, 2.6] | 0.57 |
| Breakfast prepared at home |  | 0.8 [0.4, 1.4] | 0.38 |  | 0.6 [0.3, 1.1] | 0.12 |  | 1.0 [0.5, 1.8] | 0.99 |  | 0.5 [0.3, 1.0] | 0.05 |
| Lunch prepared at home |  | 0.6 [0.3, 1.2] | 0.18 |  | 0.9 [0.5, 1.8] | 0.75 |  | 1.8 [0.9, 3.5] | 0.09 |  | 0.4 [0.2, 0.8] | 0.01 |
| Dinner prepared at home |  | 0.3 [0.1, 0.9] | 0.03 |  | 1.8 [0.8, 4.2] | 0.15 |  | 1.2 [0.5, 2.6] | 0.70 |  | 1.4 [0.6, 3.4] | 0.44 |

Associations results are adjusted betas or odds ratios and 95% confidence interval between highest (Q4) and lowest (Q1, reference) quartile of BMI genetic scores from multivariable linear or logistic regression models for PRS or GPS quartiles associations with workplace purchases and survey-derived meal habits adjusted for age, sex, seasonality, 5 principal components of ancestry, job type, education level, smoking status, and physical activity level. Higher purchasing score=healthier purchases (0-100%). Odds ratio >1 indicates more meal skipping or more meal prepared at home. Based on the biological functions of genes in or near the 97 previously identified BMI loci, the BMI_CNS_ PRS and BMInon-CNS PRS are comprised of 54 variants previously classified as CNS-related and 43 variants previously classified as non-CNS-related, respectively. *P* values are unadjusted for multiple testing.

**Abbreviations:** BMI, body mass index; CNS, central nervous system; GPS, genome-wide polygenic score; OR, odds ratio; SD, standard deviation.
