# Supplementary material for: Polygenic risk score for obesity and the quality, quantity, and timing of workplace food purchases: A secondary analysis from the ChooseWell 365 randomized trial
Source: PLoS Med. 2020 Jul 21;17(7):e1003219. doi: 10.1371/journal.pmed.1003219 (PMC7373257; doi:10.1371/journal.pmed.1003219)
Supplement: S5 Table — BMI, body mass index; GPS, genome-wide polygenic score. (DOCX) [file pmed.1003219.s007.docx]

**S5 Table.** Sensitivity analyses for BMI GPS associations with workplace purchases and self-reported meal skipping and meals prepared at home based on other *P* value thresholds (*P* =0.25; 0.50; 1.00).

|  |  | ***P* =0.19 (best fit)** | |  | ***P* =0.25** | |  | ***P* =0.50** | |  | ***P* =1.00** | |
| --- | --- | --- | --- | --- | --- | --- | --- | --- | --- | --- | --- | --- |
| **Independent SNPs, *n*** |  | 64,952 | |  | 73,412 | |  | 98,995 | |  | 126,161 | |
| **BMI variance, %** |  | 14.8 | |  | 14.8 | |  | 14.0 | |  | 13.6 | |
|  |  | Beta or OR  [95% CI] | *P* value |  | Beta or OR  [95% CI] | *P* value |  | Beta or OR  [95% CI] | *P* value |  | Beta or OR  [95% CI] | *P* value |
| **Workplace purchases** |  |  |  |  |  |  |  |  |  |  |  |  |
| Healthy purchasing score, % |  | -4.8 [-8.6, -1.0] | 0.02 |  | -4.7 [-8.4, -0.9] | 0.02 |  | -4.4 [-8.1, -0.6] | 0.03 |  | -4.5 [-8.2, -0.7] | 0.03 |
| Total purchases, units |  | 14.6 [-5.4, 34.5] | 0.16 |  | 17.3 [-2.5, 37] | 0.08 |  | 12.1 [-7.7, 32] | 0.17 |  | 12.4 [-7.5, 32.4] | 0.25 |
| Food purchases, units |  | 14.4 [-0.1, 29.0] | 0.03 |  | 16.9 [2.4, 31.3] | 0.01 |  | 15.2 [0.7, 29.6] | 0.02 |  | 16.8 [2.2, 31.3] | 0.01 |
| Beverage purchases, units |  | 0.1 [-9.1, 9.3] | 0.97 |  | 0.4 [-8.8, 9.6] | 0.97 |  | -3 [-12.2, 6.2] | 0.52 |  | -4.3 [-13.5, 4.9] | 0.30 |
| Breakfast timing, minutes |  | 15.0 [1.5, 28.5] | 0.03 |  | 10.8 [-2.6, 24.2] | 0.11 |  | 13.1 [-0.3, 26.6] | 0.06 |  | 12.8 [-0.7, 26.3] | 0.06 |
| Lunch timing, minutes |  | 6.9 [-1.8, 15.5] | 0.12 |  | 7.5 [-1.1, 16.1] | 0.09 |  | 6.1 [-2.5, 14.7] | 0.16 |  | 6.2 [-2.4, 14.9] | 0.16 |
| **Self-reported** |  |  |  |  |  |  |  |  |  |  |  |  |
| Skip breakfast |  | 1.6 [0.9, 3.0] | 0.11 |  | 1.6 [0.9, 2.9] | 0.15 |  | 1.7 [0.9, 3.1] | 0.09 |  | 1.5 [0.8, 2.8] | 0.19 |
| Skip lunch |  | 1.1 [0.6, 2.1] | 0.74 |  | 1.1 [0.6, 2.1] | 0.67 |  | 1.2 [0.6, 2.2] | 0.60 |  | 1.1 [0.6, 2] | 0.81 |
| Skip dinner |  | 1.6 [0.8, 3.3] | 0.23 |  | 1.6 [0.8, 3.4] | 0.20 |  | 1.7 [0.8, 3.5] | 0.18 |  | 1.8 [0.9, 3.9] | 0.11 |
| Breakfast prepared at home |  | 0.7 [0.4, 1.2] | 0.18 |  | 0.6 [0.4, 1.1] | 0.13 |  | 0.6 [0.4, 1.2] | 0.14 |  | 0.7 [0.4, 1.2] | 0.16 |
| Lunch prepared at home |  | 0.7 [0.4, 1.3] | 0.21 |  | 0.6 [0.3, 1.1] | 0.12 |  | 0.6 [0.3, 1.1] | 0.08 |  | 0.6 [0.3, 1.1] | 0.10 |
| Dinner prepared at home |  | 0.3 [0.1, 0.9] | 0.03 |  | 0.3 [0.1, 0.8] | 0.02 |  | 0.3 [0.1, 0.9] | 0.03 |  | 0.2 [0.1, 0.7] | 0.01 |

Associations results are adjusted betas or odds ratios and 95% confidence interval between highest (Q4) and lowest (Q1, reference) quartile of BMI GPS genetic score from multivariable linear or logistic regression models for GPS quartiles associations with workplace purchases and survey-derived meal habits adjusted for age, sex, seasonality, 5 principal components of ancestry, job type, education level, smoking status, and physical activity level. Higher purchasing score=healthier purchases (0-100%). Odds ratio >1 indicates more meal skipping or more meal prepared at home. Linkage Disequilibrium clumping and GPS generation were computed using PRSice, and the best fit genome-wide BMI GPS based on this cohort’s inverse normalized BMI encompassed 64,952 SNPs at *P* value threshold =0.19. Association *P* values are unadjusted for multiple testing.

**Abbreviations:** BMI, body mass index; GPS, genome-wide polygenic score; OR, odds ratio; SNP, single nucleotide polymorphism.
