# Supplementary material for: Polygenic risk score for obesity and the quality, quantity, and timing of workplace food purchases: A secondary analysis from the ChooseWell 365 randomized trial
Source: PLoS Med. 2020 Jul 21;17(7):e1003219. doi: 10.1371/journal.pmed.1003219 (PMC7373257; doi:10.1371/journal.pmed.1003219)
Supplement: S1 Fig — (DOCX) [file pmed.1003219.s008.docx]

**S1 Fig.** Flow chart of included “ChooseWell 365” study participants in present analysis.

397 participants of European ancestry included in present analysis

Did not consent for genotyping

(*n*= 103 excluded)

499 participants with genetic data

Related participants

(*n* = 2 excluded)

Low quality genetic samples

(*n*= 0 excluded)

Non-European ancestry participants

(*n* =100 excluded)

602 enrolled “ChooseWell 365” study participants
