# Supplementary material for: Polygenic risk score for obesity and the quality, quantity, and timing of workplace food purchases: A secondary analysis from the ChooseWell 365 randomized trial
Source: PLoS Med. 2020 Jul 21;17(7):e1003219. doi: 10.1371/journal.pmed.1003219 (PMC7373257; doi:10.1371/journal.pmed.1003219)
Supplement: S2 Fig — Count of BMI-increasing alleles for (a) BMI97, (b) BMICNS, and (c) BMInon-CNS. BMI, body mass index; CNS, central nervous system. (DOCX) [file pmed.1003219.s009.docx]

**S2 Fig.** Count of BMI-increasing alleles for a) BMI_97_, b) BMI_CNS_, and c) BMI_non-CNS_.
