# Supplementary material for: Polygenic risk score for obesity and the quality, quantity, and timing of workplace food purchases: A secondary analysis from the ChooseWell 365 randomized trial
Source: PLoS Med. 2020 Jul 21;17(7):e1003219. doi: 10.1371/journal.pmed.1003219 (PMC7373257; doi:10.1371/journal.pmed.1003219)
Supplement: Attachment — Submitted filename: R1_Comments_ChoosewellGeneticAnalysis_FINAL.docx [file pmed.1003219.s010.docx]

**Requests from the editors:

1. Please revise your title according to PLOS Medicine's style. Your title must be nondeclarative and not a question. It should begin with main concept if possible. "Effect of" should be used only if causality can be inferred, i.e., for an RCT. Please place the study design ("A randomized controlled trial," "A retrospective study," "A modelling study," etc.) in the subtitle (ie, after a colon).**

Response 1. We have revised the title. The new title is: Genetic risk for obesity and the quality, quantity, and timing of workplace food purchases: a cohort study

**2. Aim and study design should be clearly stated in the background and methods section respectively**

Response 2. We now clearly state the aim (page 4, line 59) and study design (page 4, line 63).

**3. Please provide brief participant demographics and when the study took place**

Response 3. This information has been added (page 5, line 95).

**4. Please provide participant numbers**

Response 4. Participant sample size is included (page 5, line 95).

**5. Please provide precise p-values, except when p<0.001**

Response 5. We now provide precise p-values, except when p<0.001.

**6. The last sentence of the methods and findings section must highlight a limitation of your study design or methodology**

Response 6. We have now included a limitation of our study design in the last sentence of the Abstract (page 5, line 105).

**7. Abstract Conclusions:**

*** Please address the study implications without overreaching what can be concluded from the data; the phrase "In this study, we observed ..." may be useful.**

*** Please interpret the study based on the results presented in the abstract, emphasizing what is new without overstating your conclusions.**

*** Please avoid vague statements such as "these results have major implications for policy/clinical care". Mention only specific implications substantiated by the results.**

*** Please avoid assertions of primacy ("We report for the first time....")**

Response 7. We have now updated our abstract conclusion (page 5, line 109).

**8. At this stage, we ask that you include a short, non-technical Author Summary of your research to make findings accessible to a wide audience that includes both scientists and non-scientists. The Author Summary should immediately follow the Abstract in your revised manuscript. This text is subject to editorial change and should be distinct from the scientific abstract. Please see our author guidelines for more information:** [**https://journals.plos.org/plosmedicine/s/revising-your-manuscript#loc-author-summary**](https://journals.plos.org/plosmedicine/s/revising-your-manuscript#loc-author-summary)

Response 8. We now include a short, non-technical Author Summary (page 6, line 131).

**9. Prospective analysis plan
Did your study have a prospective protocol or analysis plan? Please state this (either way) early in the Methods section.

a) If a prospective analysis plan (from your funding proposal, IRB or other ethics committee submission, study protocol, or other planning document written before analyzing the data) was used in designing the study, please include the relevant prospectively written document with your revised manuscript as a Supporting Information file to be published alongside your study, and cite it in the Methods section. A legend for this file should be included at the end of your manuscript.

b) If no such document exists, please make sure that the Methods section transparently describes when analyses were planned, and when/why any data-driven changes to analyses took place.

c) In either case, changes in the analysis-- including those made in response to peer review comments-- should be identified as such in the Methods section of the paper, with rationale.**

Response 9. We now mention our prospective analysis plan (page 9, line 234).

**10. Introduction: Please  conclude this section with a clearly outlined aim of  your study**

Response 10. We now revise the aim of the study (page 8, line 202).

**11. Methods: Please use the Methods heading instead of “subjects and methods”.**

Response 11. We now use the Methods heading instead (page 9, line 220).

**12. Since this is a secondary analysis of an RCT, please do explicitly mention this in the abstract and methods sections**

Response 12. We are explicitly mention this in the abstract (page 4, line 63) and methods sections (page 9, line 222).

**13. Results: Please provide precise p values**

Response 13. We now provide precise p values in the Results.

**14. Line 310-311- we do not permit instances of data not shown, so please provide these data or remove this sentence**

Response 14. Exact results are now presented in supplementary data.

**15. Please report your study according to the relevant guideline (**[**http://www.equator-network.org/**](http://www.equator-network.org/)**), perhaps STROBE/STREGA guideline, and include the completed checklist as Supporting Information. When completing the checklist, please use section and paragraph numbers, rather than page numbers. Please add the following statement, or similar, to the Methods: "This study is reported as per the Strengthening the Reporting of Observational Studies in Epidemiology (STROBE) guideline (S1 Checklist)." Please report your study according to the relevant guideline, which can be found here:** [**http://www.equator-network.org/**](http://www.equator-network.org/)

Response 15. We now attach a STROBE checklist.

**Reviewer #1: The authors present a very interesting study that correlates genetic risk scores for BMI with food choices. The genetic risk scores are generated in four different ways based on an existing GWAS, one genetic risk score is based on genome-wide data (GRS), while the other three scores are based on 97 genome-wide significant SNPs, which is further subdivided into 54 SNP related to the central nervous system and 43 SNPs which are not.

The design of the study is innovative and could provide important insights into how genetics influence human behaviour. The manuscript is well written and clear. Yet, I would appreciate if the authors could address my comments on the statistical analysis.**

We would like to thank the Reviewer for their favorable review of our work and their constructive feedback.

**Major comments:**

**1. “Different results depending on the risk score used: “How do the authors explain the stark difference in results between the GRS and 97 SNP risk scores as shown in Figure 2? While there are suggestive findings for GRS, the results for the 97 SNP risk score are Null.”**

Response 1. Relative to the 97 SNP risk score, the genome-wide polygenic score (GPS) captures over 6 times more variance in phenotypic BMI, which may contribute to a more robust score. For most of our GPS findings, the 97 SNP score associations are consistent in direction, suggesting that it is possible that a larger sample size may be necessary to unravel these consistent findings.

We now explain these differences between the GPS and 97 SNP risk score results in the Discussion (page 20, line 556).

**2. “Overfitting of the genome-wide PRS: How robust are the results with respect to different p-value cut-offs of the genome-wide PRS? A p-value threshold of 0.19 seems rather lenient and arbitrary. Did the authors perform any more sensitivity analysis on this p-value threshold? May the tuning of the threshold parameter in the actual dataset induce overfitting?”**

Response 2. The weighting of effect estimates for the BMI GPS is derived from an independent published study (reference 8), and thus reducing the risk of overfitting. The p-value threshold was not chosen arbitrarily, as it was determined as the best fit genome-wide BMI GPS based on this cohort’s BMI data by PRSice.

However based on the Reviewer’s comment, we now include sensitivity analyses with 3 other p-value thresholds (p =1.0, 0.50, 0.25). Methods are presented in the main text (page 14, line 404) and findings are described in Supplementary Table 5. Overall, the results remain similar, except for breakfast timing, which has a p-value 0.06.

**3. “Multiple testing: Why did the study not adjust for multiple testing? In my opinion this study would require to adjust at least for the number of outcomes examined.”**

Response 3. Considering the correlation among our outcome variables and our modest sample size, we had decided that a Bonferroni correction was likely too stringent. Yet, we agree that we need to be transparent about the multiple outcomes we have tested and the risk of chance findings. We have added this as a limitation in our Discussion (page 22, line 602).

**4. “Power calculation: Did the authors perform a power calculation prior to the study? Is the study well-powered to detect differences in food choice based on genetic risk?”**

Response 4. It is not possible to conduct power calculation for food choices as no prior genetic study has tested the unique food choice outcomes analyzed in the present study. Instead, we had first tested associations between all genetic scores and BMI in this cohort to confirm that our genetic scores are robust to detect differences in BMI (i.e., Figure 1). Although these associations do not replace formal power calculations, we believe that these findings provide support to our ability to detect associations with food choices as we expect food choices to be an endophenotype for BMI. We have added this as a limitation in our Discussion (page 22, line 604).

**5. “How representative is the sample? Supplementary Table 1 states that 80% of the participants are women, 70% have high physical activity and the average BMI is 27.9. What were the selection criteria for enrolling participants in the Choosewell study? Please discuss if there is potential selection bias in the study due to the participants not being selected at random but volunteering.”**

Response 5. MGH is a 999-bed teaching hospital in Boston, Massachusetts with over 27,000 employees who are 70% female and have a mean age of 41 years (reference 16 & 17). Employees were eligible for the study if they were between ages 20 and 75 years and used their employee badge to purchase cafeteria items at least 4 times per week for at least 6 weeks during a 12-week period prior to recruitment. Exclusion criteria included pregnancy, desire to gain weight, participation in a weight-loss study, weight loss surgery in the prior 6 months, history of an eating disorder, employment as cafeteria staff, or plan to leave MGH employment in the upcoming year.

The study participants were more likely to be female and slightly older than the average MGH workforce. Selection bias is possible due to the inclusion/exclusion criteria of the RCT and additional criteria necessary for this analysis. The sample included in our study was comprised of a relatively small sample of healthy working adults of European ancestry (restricted to avoid population stratification issues) at a large urban hospital who had volunteered to enroll in a health promotion study, and these factors may limit generalizability of our findings to other working populations and rural or non-employed people.

We’ve expanded our description of the study population (page 9, line 225) and our discussion of generalizability of our findings in the Limitation section (page 22, line 612).

**6. “Why did the authors decide to summarise the polygenic scores into quartiles and present results as the comparison between highest and lowest quartile? How do the results look like when including the exact scores as quantitative predictors into the model?”**

Response 6. We decided to present results for genetic risk score quartiles rather than a continuous per allele score to make it easier to interpret and present the study results.

We now present linear associations for our genetic risk scores in Supplementary Table 3. Associations are presented per risk allele (for all scores except GPS) or per standard deviation (for GPS). Overall, linear associations remain similar to findings from quartiles (page 18, line 507).

**Minor comments:
--------------------
1. Supplementary Figure 1. Please put "" around "ChooseWell 365", otherwise this may read as 365 study participants.**

Response 1. We now included “” around ChooseWell 365 consistently throughout the manuscript and Supplementary Figure 1.

**2. Supplementary Table 2. Explain "highly expressed in the CNS" in the legend.**

Response 2. We now explain this in the legend of Supplementary Table 2.

**Reviewer #2: “The authors present a secondary analysis(?) of an impressive trial, where participants health behaviour manipulated. The dataset is rich as objective food purchases have been measured, along with genetics. I would be very intrigued to read analysis of the phenotypic BMI in relation to the objective eating study variables. The PGS just seems to serve as a fancy method, but I think using PGS is an overkill - it makes associations very small and ultimately does not deliver any extra interpretative value.”**

We would like to thank the Reviewer for their feedback on our work and their recognition of the importance of our trial. While associations related to the genetic scores may be modest, they suggest for the first time that genetic variants previously established for BMI may influence food purchasing behavior and therefore identify potential targets for research to develop personalized interventions to prevent weight gain.

**1. “The study seems as a secondary analysis, as although the study is a trial, the manipulation is not being analysed. It would be helpful to have a reference to the paper analysing primary outcomes of the study, if available. The clinical trial outlines a moderating role of the PGS. This does not seem to be tested here. Please outline clearly, whether you followed the clinical trials protocol in this analysis or conducted exploratory additional analyses. Both approaches are ok, this just needs to be clear.”**

Response 1. The present study is a secondary analysis of baseline data from the “Choosewell 365” health promotion randomized control trial. The trial just completed 2-year follow-up and the primary outcome paper is under development. The analysis for the current study does not utilize any post-randomization data and therefore is not related to the clinical trial analysis plan. We have clarified the study design and our analytical approach in the Abstract (page 4, line 63) and Methods (page 9, line 222).

**2. “The study's goals fall short likely due to smallish sample size for these goals. This is exemplified by the use of quartiles throughout the paper, which highlight associations that likely do not emerge when using PGS-s as linear predictors. This approach has been previously critiqued in relation to polygenic scores. See this blog for accessible overview and further materials.** [**https://discourse.datamethods.org/t/discussion-of-polygenic-prediction-of-weight-and-obesity-trajectories-from-birth-to-adulthood/1585**](https://discourse.datamethods.org/t/discussion-of-polygenic-prediction-of-weight-and-obesity-trajectories-from-birth-to-adulthood/1585)  **. If the authors wish to use quartiles, they should justify their choice in relation to this critique.”**

Response 2. Reviewer 1 also requested presenting linear associations (see R1 comment #6 above). We now present linear associations for our genetic risk scores in Supplementary Table 3. Associations are presented per risk allele (for all scores except GPS) or per standard deviation (for GPS). Overall, linear associations remain similar to findings from quartiles (line 18, page 507).

**3. “I invite authors to think through, what is the message and scientific question they want to deliver. If the goal is to understand how obesity influences food choices and reactiveness to the trial, then I would just use phenotypic obesity. This allows using a bigger sample, and also having bigger statistical effects. I would be very interested in learning the phenotypic results!”**

Response 3. It remains unknown if genetic risk for obesity directly influences food choice behaviors and dietary intake, and therefore our objective for this study was to understand whether genetic risk for obesity is associated with quality, quantity, or timing of food choices, each of which may influence the relationship between the obesity risk alleles and obesity. A secondary objective was to examine whether CNS-related obesity genetic risk may be more likely to influence food behavioral choices than the non-CNS component. Thus our primary exposure of interest was the genetic variants previously identified for obesity rather than phenotypic BMI. Future analyses from this trial will leverage phenotypic BMI data and 1-year and 2-year trial outcomes.

**4. “Generally speaking, BMI_PGS-phenotype associations reproduce known BMI_phenotype-phenotype associations. So you could just study phenotypic associations and conclude that this is likely how genetic BMI plays out. Perhaps you could try reproducing the strongest phenotypic BMI- eating associations with a BMIpgs?”**

Response 4. Our hypothesis was that genetic variants for BMI influence BMI through food choices and therefore food choices might partially mediate the relationship between these genetic variants and BMI. While genome-wide polygenic risk score associations may reflect phenotypic associations, we were particularly interested in the possible influence of previously determined genetic variants for obesity, including CNS and non-CNS related variants separately, on food purchases and behaviors. These findings can help determine whether genetic predisposition to obesity also predisposes to certain food choice behaviors, and thus informing future trials investigating whether lifestyle interventions targeting specific food choice behaviors can then modify genetic risk for weight gain and obesity.

**5. “If you want to make claims between BMIcns- and BMInon-cns, you need to test the statisical difference in effect sizes.** [**http://comparingcorrelations.org/**](http://comparingcorrelations.org/)**”**

Response 5. We now calculate statistical heterogeneity between CNS-related and non-CNS-related results for all outcomes. These results have been added to the Results (page 17, line 471 and page 18, line 504) and Figures 3 and 5.

**6. “Polygenic scores can also be used in certain conditions to infer causality. But I think that you need a larger sample size to do this kind of research.
Doi.org/10.1002/sim.6835”**

Response 6. Indeed, our modest sample size limits our ability to infer potential causal links in Mendelian randomization analyses. We have included this as a limitation in the Discussion (page 22, line 607).

**Reviewer #3: “This study addresses a more relevant aspect in the knowledge of determining factors of obesity that has also been scarcely described as is the choice of food. The work is well written and the results presented, below with some comments:”

1.”The authors mention the fact that is in workplace, it could be also interesting if they could discuss about what kind of food is available in those cafeteria and if that could be a bias.”**

Response 1. The cafeterias at MGH are open 5 or 7 days per week, depending on site. All are open in the mornings through afternoons for breakfast and lunch, with more limited availability in the evenings and overnight. More than 1200 different food items are available over the course of a day including meals and entrees (hot prepared meals, pre-made sandwiches and salads, and pizza), a large salad bar, snacks and desserts, as well as hot and cold beverages. Options are distributed roughly evenly between those labeled green (34%), yellow (37%), or red (29%) (reference 17). The average costs of red, yellow, and green items were comparable for beverages, entrees, and snacks/side items, and items across a range of prices were available in each color category.

We have included this information in the Methods section (page 10, line 264).

**2. ”The authors mention that the collection was made three months retrospectively, is that because it was part of another study or why it is measured retrospectively? In that sense the authors calculated the average?”**

Response 2. In order to associate genetic risk scores with habitual workplace purchasing behavior (i.e., behavior at baseline and prior to trial intervention), we retrospectively accessed cafeteria sales data for each consented participant. These data are available from the hospital’s cafeteria sales database. The 3-month data were averaged for each participant. We used 3 months as a period of time that would represent typical and habitual purchases and avoid abnormalities due to short term vacations or work schedule changes. We now clarify this in the Methods section (page 10, line 254).

**3. “It would be interesting if the authors could characterize the types of food available in the cafeteria, in addition to saying the traffic lights.”**

Response 3. As described in our response to comment 1 above, more than 1200 different food items are available over the course of a day including meals and entrees, a large salad bar, snacks and desserts, as well as hot and cold beverages. Options are distributed roughly evenly between those labeled green (34%), yellow (37%), or red (29%) (reference 17). The average costs of red, yellow, and green items were comparable for beverages, entrees, and snacks/side items, and items across a range of prices were available in each color category. We have included this information in the Methods section (page 10, line 264).

**4. “Although in complementary material they indicate the genes evaluated, it is interesting that in the discussion they could deepen into this and how some genes could effectively modulate aspects of food choice, put it in more concrete terms on the mechanism.”**

Response 4. While the precise mechanism of how BMI-related genes may influence purchasing behavior cannot be elucidated from our analysis, we have now expanded our explanation of possible underlying biological pathways that may per pertinent to our findings. Specifically, anorexigenic and orexigenic signaling pathways (*BDNF*), nutrient preference such as carbohydrate (*RARB*) and fat (*ADH1B*), and energy homeostasis (*MTCH2*), all constitute mechanisms that may influence purchasing behavior. However, the precise biological role of most BMI loci remain to be elucidated.

We have included this this in our Discussion section (page 20, line 546).
