# Supplementary material for: Polygenic risk score for obesity and the quality, quantity, and timing of workplace food purchases: A secondary analysis from the ChooseWell 365 randomized trial
Source: PLoS Med. 2020 Jul 21;17(7):e1003219. doi: 10.1371/journal.pmed.1003219 (PMC7373257; doi:10.1371/journal.pmed.1003219)
Supplement: Attachment — Submitted filename: R2_Comments_ChoosewellGeneticAnalysis_FINAL.docx [file pmed.1003219.s011.docx]

**Requests from Editors:**

**1. Suggest title is tempered as you are not directly measuring genetic risk? In addition, the study descriptor might be better as ‘a secondary analysis from the ChooseWell 365 randomised trial’.**

Response 1. Based on our discussion with the Editor, we have revised the title. The new title is: Polygenic risk score for obesity and the quality, quantity, and timing of workplace food purchases: a secondary analysis from the ChooseWell 365 randomised trial

**2. Please provide a table with demographic information of participants**

Response 2. The demographic information of the ChooseWell 365 participants was previously provided as a Supplementary Table 1. We now move this table to the main text and provide is as a Table 1 instead.

**3. Please consider revising “influencing” to perhaps “associated with” as this is an observational study “We also assessed whether higher CNS-related obesity genetic risk that may be more likely to influence food behavioural choices than the non-CNS component”. The same goes for where you mention “causal in pathway”. I don’t think the study design or results allow this extrapolation, so please use more cautious language throughout.**

Response 3. We have revised “influence” to “associated with” in the sentence (page 8, line 165) and have removed all mention of causal implications of our findings (page 6, line 116; page 21, line 426; and page 23, line 481;) **4. It appears that your STROBE checklist was not included in the revised manuscript and it was not called out in the methods section. Please do so, as requested previously. We ask that you don’t use line or page numbers as these are likely to change during publication**

Response 4. We apologize for not attaching the STROBE checklist previously. We have now added it and call it out in the Methods section (page 9, line 166).

**5. In the methods, in addition to simply mentioning that you had a prospective analysis plan, please provide this analysis plan as an SI file and also provide a call out to the file in the methods section. As stated previously “If a prospective analysis plan (from your funding proposal, IRB or other ethics committee submission, study protocol, or other planning document written before analyzing the data) was used in designing the study, please include the relevant prospectively written document with your revised manuscript as a Supporting Information file to be published alongside your study, and cite it in the Methods section. A legend for this file should be included at the end of your manuscript”.**

Response 5. We now attach our prospective analysis plan as a Supporting Information file 1 and reference it in the Methods (page 9, line 183) and have included a legend for this file at the end of the manuscript (page 31).

**6. Please add unaccounted confounding as a limitation in your discussion, as there are several confounders linked to food choice and not all can be measured in an observational setting.**

Response 6. We now mention unaccounted confounding as a limitation on page 23, line 473: “Despite our covariate adjustment, we recognize that a general weakness of observational studies is a risk of bias due to residual confounding.”

**Comments from statistical reviewer:

Regarding multiple testing, I perfectly agree with the authors that a Bonferroni correction would be too strict. Nonetheless I think it is necessary to perform some type of multiple testing correction in order to prevent false positive findings.
 
The R-function p.adjust():**
[**https://www.rdocumentation.org/packages/stats/versions/3.6.2/topics/p.adjust**](https://www.rdocumentation.org/packages/stats/versions/3.6.2/topics/p.adjust) **offers a wide range of possible alternatives to the Bonferroni correction. The method by Benjamini & Yekutieli (2001) (option: "BY") allows for correlated tests:
Benjamini, Y., and Yekutieli, D. (2001). The control of the false discovery rate in multiple testing under dependency. Annals of Statistics, 29, 1165--1188. 10.1214/aos/1013699998.
 
Alternatively, there is the concept of the "effective number of tests" which estimates the actual number of independent tests from a set of correlated tests. It is implemented in R in the function meff() in the poolr package:**
[**https://cran.r-project.org/web/packages/poolr/poolr.pdf**](https://cran.r-project.org/web/packages/poolr/poolr.pdf) **For the number of effective tests, the method by Nyholt is the most popular option:
Nyholt, D. R. (2004). A simple correction for multiple testing for single-nucleotide polymorphismsin linkage disequilibrium with each other. American Journal of Human Genetics, 74(4), 765-769.**

Statistical Reviewer Response: Based on our discussion with the Editor, we now provide unadjusted and adjusted P values. Adjusted P values were calculated using the R-function p.adjust() using the false discovery rate method. We now describe our approach in the Methods (page 15, line 323), present findings in the Results (page 19, line 382; and Supplementary Table 2), and discuss this as a limitation in our Discussion (page 23, line 464).
